# A machine learning-based classification approach on Parkinson’s disease diffusion tensor imaging datasets

**DOI:** 10.1186/s42466-020-00092-y

**Published:** 2020-11-10

**Authors:** Jannik Prasuhn, Marcus Heldmann, Thomas F. Münte, Norbert Brüggemann

**Affiliations:** 1 Department of Neurology, Institute of Neurogenetics, University of Lübeck, Ratzeburger Allee 160, 23538 Lübeck, Germany; 2 Department of Neurology, University Medical Center Schleswig-Holstein, Campus Lübeck, Ratzeburger Allee 160, 23538 Lübeck, Germany; 3 Institute of Psychology II, University of Lübeck, Ratzeburger Allee 160, 23538 Lübeck, Germany

**Keywords:** Parkinson’s disease, DTI, Machine learning, Substantia nigra, Neuroimaging

## Abstract

**Introduction:**

The presence of motor signs and symptoms in Parkinson’s disease (PD) is the result of a long-lasting prodromal phase with an advancing neurodegenerative process. The identification of PD patients in an early phase is, however, crucial for developing disease-modifying drugs. The objective of our study is to investigate whether Diffusion Tensor Imaging (DTI) of the Substantia nigra (SN) analyzed by machine learning algorithms (ML) can be used to identify PD patients.

**Methods:**

Our study proposes the use of computer-aided algorithms and a highly reproducible approach (in contrast to manually SN segmentation) to increase the reliability and accuracy of DTI metrics used for classification.

**Results:**

The results of our study do not confirm the feasibility of the DTI approach, neither on a whole-brain level, ROI-labelled analyses, nor when focusing on the SN only.

**Conclusions:**

Our study did not provide any evidence to support the hypothesis that DTI-based analysis, in particular of the SN, could be used to identify PD patients correctly.

## Background

Diffusion tensor imaging (DTI) has been proposed for analyzing microstructural integrity not only of white but also grey matter. However, the use of DTI to observe, e. g., subcortical grey matter changes is currently under debate [15]. Whether microstructural alterations of the whole brain, regions of interests (ROI)-labeled grey matter, or the substantia nigra (SN) can be detected applying diffusion metrics in Parkinson’s disease patients (PD) is still unclear. The significance of several previous DTI studies in PD is limited due to small sample sizes and by the fact that specific regions of interests were delineated manually for the extraction of diffusion metrics. Besides, studies that were able to demonstrate significant group differences have also shown a relevant overlap of diffusion metrics between PD patients and healthy controls, which undermines the potential diagnostic use. Machine learning-based (ML) models might help to detect subtle alterations of diffusion metrics, by their multivariate nature and by the integration of different imaging modalities, and to improve their diagnostic use subsequently. The aforementioned practice also hindered the translation into clinical practice [19]. Our study hypothesizes that ML algorithms and the application of a suitable sub-cortical atlas for the elderly population can be used to distinguish between PD patients and age- and gender-matched healthy controls in a standardized and therefore potentially more sensitive manner [5]. Computing algorithms like binary support vector machines (bSVM) or multiple-kernel learning (MKL) provide suitable and promising tools to address classification problems based on neuroimaging data [18]. Advancements in the multivariate interpretation of neuroimaging data have already been proven useful in a plethora of neuropsychiatric [16] and neurodegenerative diseases [11, 12]. Besides, the employment of machine-learning algorithms to Parkinson’s disease datasets has offered unique advancements in interpreting distinct neuroimaging modalities [3, 4, 20, 23]. MKL also yields the opportunity to concatenate different imaging modalities. This is of particular interest as distinct diffusion metrics are meant to resemble different histopathological hallmarks of neurodegeneration [22].

## Methods

DTI datasets of 162 PD patients (age: 63.9 ± 9.3 years; gender: 34.2% female; disease duration: 6.5 ± 4.1 months; mean MDS-UPDRS-III: 13.9 ± 2.1; mean Hoehn and Yahr stadium: 1.2 ± 0.3) and 70 age and gender-matched healthy controls (HC) (age: 62.1 ± 10.1 years; gender: 34.9% female) were analyzed. This study used human subject recordings chosen from the Parkinson’s Progression Marker Initiative (PPMI) database. The PPMI dataset was published open-access with a positive ethics statement of the responsible authorities. Therefore, additional ethics committee approvals do not apply to this study. DTI-MR sequences were acquired on a Siemens 3 T TIM Trio scanner using a 12-channel matrix head coil and a two-dimensional echo-planar DTI sequence (TR/TE = 900/88 ms, flip angle = 90°, voxel size = 2 × 2 × 2 mm^3^, 72 slices, 64 gradient directions with a b-value of 1000 s/mm^2^). In addition, a non-gradient volume (b = 0 s/mm^2^) was acquired as well. Further details of the PPMI image acquisition protocol can be seen online (http://www.ppmi-info.org/wp-content/uploads/2017/06/PPMI-MRI-Operations-Manual-V7.pdf). We performed pre-processing by using the PANDA-toolbox (v1.3.1) in Matlab 2018b, including normalization to standard space (via FMRIB58_FA template, 2 mm × 2 mm × 2 mm voxel size) [6]. In addition to conventional diffusion metrics (FA, MD, AD, and RD), we calculated local diffusion homogeneity (LDH) as another measure of microstructural white matter integrity. For the interpretation of DTI images, we calculated the following standard diffusion metrics based on the three-dimensional diffusion of water as a function of spatial location: Fractional Anisotropy (FA) is a summary measure for interpreting microstructural integrity. Mean Diffusivity (MD) is a measure of the cell membrane density. It is, therefore, sensitive for cellularity, edema, and necrosis of investigated tissue. Axial Diffusivity (AD) decreases in axonal injury. Radial Diffusivity (RD) increases in de- or dysmyelination of axons. A concise review article on the interpretability of diffusion metrics to investigate microstructural grey and white matter changes are described in a review article by Alexander et al. [1]. Local diffusion homogeneity (LDH) is another diffusion metric that is specifically relevant to assess tissue homogeneity based on neighboring voxels [9]. We computed LDH for 6, 18, and 26 neighboring voxels using Spearman’s Rank Correlation coefficient (06LDHs, 18LDHs, and 26LDHs) and Kendall’s coefficient concordance (06LDHk, 18LDHk, and 26LDHk) [9]. Voxel-wise whole-brain analysis was performed using the FM-RIB58_FA template. We performed ROI-labeled analyses based on the well-established AAL atlas [21]. To further increase the signal-to-noise ratio, we additionally performed classification after masking of the SN using the ATAG atlas for the elderly population [10]. The datasets were classified through bSVMs (for single modalities) as well as MKL (for concatenated modalities). Ten-fold cross-validation (CV) and nested (leave one subject out) hyperparameter optimization as implemented in the PRoNTo-Toolbox (v2.1) [18]. The determination of relevant bSVM and MKL parameters (such as the applied L1 regularization method or the nested hyperparameter optimization) is following standard practice and is extensively described in the publications of Schrouff et al. [17, 18]. Age, gender, and total intracranial volume were used as covariates. Balanced Accuracy (BA) and area under the curve of the receiver-operating characteristic curve (ROC-AUC) were calculated to assess classification performance and were compared to random permutation testing (against 10.000 permutations).

## Results

**Fig. 1:**
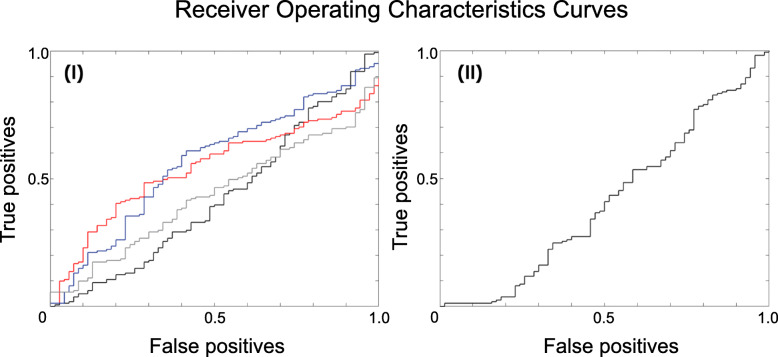
ROCs for (I) FA (red), MD (black), RD (blue), and AD (grey) each as a single modality (bSVM) and (II) as concatenated modalities (MKL). Based on our results, the ROCs are indicating no substantial diagnostic value. Further, the concatenation of DTI modalities yields no additional information for this classification problem

**Fig. 2:**
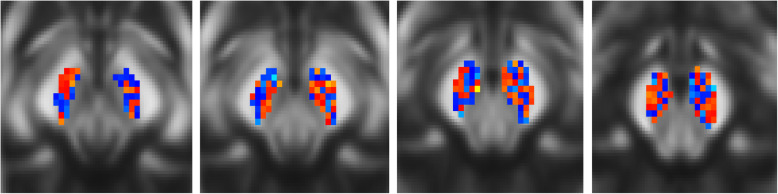
Weight maps of FA bSVM (shown on axial midbrain slices). The pattern indicates random weighting of FA values for the purpose of classification (comparable to the other investigated diffusion metrics and the MKL, data not shown here). Former studies demonstrated altered diffusion metrics in the occipital portions of the SN [13]. However, interpretability is limited e.g. due to the small ROI size

The application of the bSVM on the various types of diffusion metrics revealed that there are no significant differences concerning the BA or the ROC-AUC for voxel-wise whole-brain or AAL-based ROI-labeled analyses (data not shown here). As most studies suggest, diffusion metrics are most likely altered in the SN of PD patients, making the SN the region of highest interest to increase the signal-to-noise ratio for classification [19]. Therefore, further analyses focused on the diffusion metrics of the masked SN and will be reported in the following (see Fig. 1). Again, there were no significant differences regarding BA or ROC-AUC: FA (BA: 47.83% ROC-AUC: 0.42); MD: (BA: 50.00%, ROC-AUC: 0.54); AD: (BA: 50.00%, ROC-AUC: 0.44); RD: (BA: 50.00%, ROC-AUC: 0.56); 06LDHs: (BA: 49.47%, ROC-AUC: 0.54); 18LDHs (BA: 56.64%, ROC-AUC: 0.57); 26LDHs (BA: 53.14%, ROC-AUC: 0.53); (BA: 55.03%, ROC-AUC: 0.58); 06LDHk (BA: 55.25%, ROC-AUC: 0.52); 18LDHk (BA: 53.14%, ROC-AUC: 0.53); 26LDHk (BA: 51.80%, ROC-AUC: 0.52). The results also indicate that the concatenation of diffusion metrics via MKL did not add any relevant information to improve the overall classification performance: FA + MD + AD+RD (BA: 49.44%, ROC-AUC: 0.41); 06LDHs + 18LDHs + 26LDHs (BA: 56.15%, ROC-AUC: 0.60); 06LDHk + 18LDHk + 26LDHk (BA: 58.12%, ROC-AUC: 0.52). An overview on provided diagnostic performances in displayed in the Table 1. The comparison to random permutation testing showed that the classifications, as mentioned above, did not outperform pure chance. Additionally, calculated weight maps are indicating a random weighting distribution of voxels within the SN used for the respective classifications (see Fig. 2), which is in contrast to previously reported changes of the dorsolateral portion of the SN (i. e., the nigrosome-1) [13].

**Table 1:** Overview of diagnostic performances of single modalities (bSVM) and concatenated modalities (MKL) for the SN

bSVM					MKL				
	BA [%]	ROC-AUC	Sens [%]	Spec [%]		BA [%]	ROC-AUC	Sens [%]	Spec [%]
*FA*	47.8	.42	47	48	*FA + MD + AD + RD*	49.4	41	44	60
*MD*	50.0	.54	55	42	*06LDHs + 18LDHs + 26LDHs*	56.1	60	54	56
*AD*	50.0	.44	40	47	*06LDHk + 18LDHk + 26LDHk*	58.1	52	56	41
*RD*	50.0	.54	48	41					
*06LDHs*	49.4	.54	40	44					
*18LDHs*	56.6	.57	49	41					
*26LDHs*	53.1	.53	51	60					
*06LDHk*	55.2	.52	51	56					
*18LDHk*	53.1	.53	60	63					

Besides BA and ROC-AUC. Sens and Spec are listed to enhance the transparency of reported ROC-AUC results

*AD* Axial diffusivity, *BA* Balanced accuracy, *bSVM* Binary Support vector machine, *FA* Fractional anisotropy, *LDH* Local diffusion homogeneity, *MD* Mean diffusivity, *MKL* Multiple-kernel learning, *RD* Radial diffusivity, *ROC-AUC* Receiver operator characteristics area under the curve, *Sens* Sensitivity, *Spec* Specificity, *SN* Substantia nigra

## Discussion

In this study, we demonstrated a standardized and systematic approach to potentially attain the individual discrimination of PD patients from healthy controls using DTI datasets. This approach comprised the pre-processing of the data, the automatized selection of appropriate features, and the subsequent classification. Atkinson-Clement, Pinto, Eusebio, and Coulon [2] already stated that “[…] they did not observe a PD induced reduction of nigral FA” but also that “this observation is in contrast with some recent publications claiming very high diagnostic accuracy, but [are] well in line with other reports showing small or no PD induced nigral FA decrease”. A meta-analysis also did “not support nigral DTI metrics as a useful diagnostic marker of PD” [19]. Our results are supporting the aforementioned lack of evidence and should put discussions about the diagnostic use of diffusion metrics in PD patients to rest. The negative results of our study most likely reflect the lacking suitability of diffusion metrics to investigate SN-related microstructural alterations in PD. The interpretation of our findings within the scope of differing DTI acquisition schemes and MRI scanner hardware is challenging. However, a multicenter validation study by the authors of Fox et al. [7] stated high intersite-concordance for applied DTI metrics on different scanner hardware (3 T magnetic field strength). ML-algorithms are a more standardizable and sensitive method to increase diagnostic accuracy and to disentangle the overlap of diffusion metrics other groups reported, which were only using voxel-wise mass-univariate or manually extracted diffusion metrics for subsequent analysis. The multivariate, compared to mass-univariate, approach and the additional concatenation of modalities should enhance the discriminatory, and therefore, diagnostic accuracy substantially. The lack of significant findings despite a larger sample size and a more sensitive and sophisticated approach in this study are further supporting the view that traditional diffusion metrics are indeed missing any diagnostic use. Whether DTI can be used to map individual disease progression remains, to this point, elusive. Further methodological improvements of diffusion-based imaging might improve diagnostic accuracy and might, therefore, cause a reconsideration of our current conclusion. However, the current MRI acquisition and analysis paradigms of DTI measures are not of any use for investigating grey matter alterations in PD. Further studies without substantial methodological improvements will most likely not result in potentially translatable advancements in improving diagnostic accuracy or patient care. Recent research studies which revealed that the use of free-water corrected diffusion maps for the analysis of tissue alterations might provide the opportunity for fostering the diagnostic accuracy based on this dataset [14]. However, ML analyses of neuroimaging data is a fruitful approach in supporting clinical decision making and will be more frequently applied in the future [8]. The objective of our study was to investigate the role of ML-based algorithms on diffusion metrics to identify PD patients correctly. Our study did not provide any evidence to support the hypothesis that DTI-based analysis, in particular of the SN, could be used to resolve the issue of correctly classifying study participants independent of the phenotype. An advantage of our methodology is that by calculating weighting maps, we can additionally validate our findings: Previous literature stated that the dorsolateral parts of the SN are the ones that are particularly affected at the beginning of the disease [19]. Weighting maps should indicate the higher relevance of these specific areas for classification performance (which is in contrast to our findings, see Fig. 2). Here, this advantage is of even higher importance as further partitioning of the SN appears, within the scope of the already small region and the present image resolution, not to be feasible.

## Conclusion

Our findings are well in line with previous publications using conventional analyses. Further studies without substantial methodological improvements (e. g., utilizing more complex diffusion models) will most likely not result in potentially translatable advancements in improving diagnostic accuracy or patient care.

## Data Availability

The proposed study has been performed on publicly-available data obtained from the Parkinson’s Progression Marker Initiative (PPMI). Study analyses were performed following the PPMI Data Use Agreement (http://www.ppmi-info.org/documents/ppmi-data-use-agreement.pdf).

## Abbreviations

AAL: Automated Anatomic Labeling
AD: Axial diffusivity
ATAG: Atlas of the basal ganglia
BA: Balanced accuracy
bSVM: Binary support vector machine
CV: Cross-validation
DTI: Diffusion tensor imaging
FA: Fractional anisotropy
HC: Healthy controls
LDH: Local diffusion homogeneity
MD: Mean diffusivity
MKL: Multiple-kernel learning
ML: Machine learning
MRI: Magnetic Resonance Imaging
n/a: Not applicable
PD: Parkinson’s disease
PPMI: Parkinson’s Progression Marker Initiative
RD: Radial diffusivity
ROC-AUC: Receiver operator characteristics area under the curve
ROI: Region of interest
SN: Substantia nigra
